# Tourette syndrome and learning disabilities

**DOI:** 10.1186/1471-2431-5-34

**Published:** 2005-09-01

**Authors:** Larry Burd, Roger D Freeman, Marilyn G Klug, Jacob Kerbeshian

**Affiliations:** 1 Department of Pediatrics, University of North Dakota School of Medicine and Health Sciences, Grand Forks, North Dakota, USA; 2 Department of Pediatrics, University of British Columbia, Vancouver, British Columbia, Canada; 3 Department of Neuroscience, University of North Dakota School of Medicine and Health Sciences, Grand Forks, North Dakota, USA

## Abstract

**Background:**

Tourette Syndrome (TS) is a neurodevelopmental disorder of childhood. Learning disabilities are frequently comorbid with TS. Using the largest sample of TS patients ever reported, we sought to identify differences between subjects with TS only and subjects with TS and a comorbid learning disability.

**Methods:**

We used the Tourette Syndrome International Consortium database (TIC) to compare subjects with comorbid Tourette Syndrome and learning disabilities (TS + LD) to subjects who did not have a comorbid learning disability (TS - LD). The TIC database contained 5,500 subjects. We had usable data on 5,450 subjects.

**Results:**

We found 1,235 subjects with TS + LD. Significant differences between the TS + LD group and the TS - LD group were found for gender (.001), age onset (.030), age first seen (.001), age at diagnosis (.001), prenatal problems (.001), sibling or other family member with tics (.024), two or more affected family members (.009), and severe tics (.046). We used logistic modeling to identify the optimal prediction model of group membership. This resulted in a five variable model with the epidemiologic performance characteristics of accuracy 65.2% (model correctly classified 4,406 of 5,450 subjects), sensitivity 66.1%, and specificity 62.2%.

**Conclusion:**

Subjects with TS have high prevalence rates of comorbid learning disabilities. We identified phenotype differences between the TS - LD group compared to TS + LD group. In the evaluation of subjects with TS, the presence of a learning disability should always be a consideration. ADHD may be an important comorbid condition in the diagnosis of LD or may also be a potential confounder. Further research on etiology, course and response to intervention for subjects with TS only and TS with learning disabilities is needed.

## Background

Tourette Syndrome (TS) is a complex developmental disorder defined by the childhood onset of motor and vocal tics with a longitudinal outcome of gradual improvement in most subjects [1-4]. The disorder is associated with increased prevalence rates of comorbid disorders, the most common of which is attention-deficit hyperactivity disorder (ADHD) [5,6]. Learning disabilities (LD) and obsessive-compulsive disorder (OCD) or obsessive and compulsive behaviors (OCB) are also common [7-10].

In previous work we have demonstrated that over time the presence of comorbidity is an important factor in syndromal severity and in the level of impairment from the disorder [11]. While several disorders have been demonstrated to occur as a manifestation of the broad TS phenotype (OCD, ADHD) the role of several other conditions is currently a contentious issue in the definition of that broad phenotype [8-10,12]. Previous research has demonstrated increased prevalence of LD in subjects with TS but the role of LD as a manifestation of the broad TS phenotype is not yet settled [8,10,13-25].

Previous research on TS and comorbid LD has relied on relatively small samples usually selected from 1 or 2 clinic sites [13-20,22-26]. The limitations of small sample size and selected catchments for these studies have led to concerns about the generalizability of the results of these studies. In order to minimize these limitations, we have elected to utilize a large international population of cases of TS and TS with comorbid LD to examine differences in subjects with TS without a comorbid learning disability and subjects with TS and a comorbid learning disability. We utilized data from the Tourette Syndrome International Consortium (TIC) to examine differences between subjects with Tourette Syndrome and learning disabilities (TS + LD) and subjects with Tourette Syndrome who did not have a comorbid learning disability (TS - LD). We have utilized data from this consortium for multiple other studies of TS including comorbid TS and pervasive developmental disorders, prediction of tic severity, and hereditary factors in tic severity [27].

## Methods

The study population was comprised of consecutive subjects entered into the database since its inception. The 5,500 subjects in this study include the 3,500 subjects previously reported in the paper by Freeman and colleagues [28].

### Registry reporting sites

Thirty-six sites have over 50 subjects and seventeen sites have over 100 subjects. Twenty-four sites have less than 50 cases and 19 are currently inactive. The geographic distribution of the consortium cases was: Canada 40.6%, United States 22.6%, Europe 25.1%, Middle East 3.6%, South America 1.8%, Asia 3.0%, Australia 3.0%, and Africa 0.3%. The clinicians who submit cases to the registry are either physicians (nearly all) or psychologists.

### Subject selection

All subjects entered in the registry met the criteria for TS from the Tourette Syndrome Classification Study Group [29]. Each subject was reported utilizing a structured reporting format [see Additional file 1] to assure comparability of the data. A learning disabilities diagnosis entered into the registry was inclusive of specific learning disorders as defined in the DSM-IV, through the less precise and less verifiable category of learning disorders NOS [30]. The diagnosis of LD would only rarely include individuals with mental retardation (MR). In this paper we excluded the few subjects with both LD and MR. The TIC database does not have data on the proportion of subjects diagnosed with LD after psychometric testing or the proportion where LD was a clinical diagnosis or both.

Subject data were then forwarded to the consortium where each case was reviewed for inconsistencies prior to entry into the database. If errors were identified or suspected, the case file was returned to the clinical site for review. This data is not verified beyond the identification of errors in either data entry from the submitted form (data entry control procedures are utilized to minimize these errors) or unless an error is detectable by the field entry restriction values for each variable.

We utilized the method of Spady et al. for management of summary data [31]. As in most reported cohorts we were not able to detect diagnostic error. To minimize the potential impact of errors we do not report values from individual clinical sites. Thus, the results represent pooled data from multiple sites to reduce any potential impact from systematic or inadvertent error from any one site. This data pooling increases accuracy but does so by obscuring between site differences and as a result decreases precision.

### Statistical analysis

For this study we had usable data on 5,450 subjects. Continuity corrected Chi-Square was used to test the association between gender, age of onset, age first seen, age diagnosed, clinician type, perinatal problems, heredity of TS, severity of TS, and fourteen comorbidities by group (TS + LD) and (TS - LD). Since LD was used to define one of the groups in this study LD was not counted as a comorbid disorder in the study. Thus, the variable comorbidity is comprised of all other comorbid disorders available from the dataset. Observations with missing values were deleted for each univariate analysis. After completion of the univariate analysis, we used logistic regression modeling to identify the optimal set of prediction variables to predict group membership TS + LD. We used the epidemiologic performance characteristics of accuracy, sensitivity and specificity to select a final logistic model.

## Results

Of the 5,450 subjects with TS, the TS + LD group was comprised of 1,235 subjects (22.7%) while the TS - LD group had 4,215 (76.3%) subjects. In the TS - LD group, 3,774 subjects (69.2%) had other comorbid conditions. Four hundred and forty-one patients of the TS-LD group (8.1%) had no comorbid disorders or conditions. In the TS + LD group the average number of comorbidities other than LD was 3.04 (s.d. 2.07). The analysis includes all the variables included in the database. The average age of onset of TS was 6.37 years (s.d. = 2.82) and was determined by parental report of tic onset. The average age of diagnosis was 13.43 years (s.d. = 10.0). The TIC Registry population was 81.4% male, while 19.3% had perinatal problems and 53.9% had at least one family member with a history of tics or TS.

Table 1 shows variables with significant associations for TS + LD. Subjects with TS + LD had an increased proportion of males (p < .001), and have an age of onset of TS before eight years of age (p = .030). They also were first seen before 18 years of age (p < .001) and were diagnosed before they were thirteen years of age (p < .001). The average age of onset for those with TS + LD was 6.14 (s.d. = 2.56) and was comparable to 6.44 years (s.d. = 2.88) for those with TS - LD. The average age first seen for those with TS + LD was 12.5 (s.d. = 7.5), while it was over three years later (mean = 15.7, s.d. = 11.6) for those with TS - LD. The average age for diagnosis in TS + LD was also three years earlier (mean = 11.4, s.d. = 7.1) compared to those with TS - LD (mean = 14.0, s.d. = 10.6). Seventy-four percent of the cases were diagnosed by a psychiatrist and 19% were diagnosed by a neurologist (p < .001).

**Table 1:** Between group comparisons in 5,450 subjects with Tourette Syndrome and Learning Disabilities (TS + LD) and Tourette Syndrome without learning disabilities(TS-LD) by gender, age, perinatal problems, and family history of tics.

	TS + LD		TS - LD		
	n	(%)	n	(%)	p
Gender					
Female	159	(12.9)	857	(20.3)	<.001
Male	1,076	(87.1)	3,355	(79.7)	
Age of Onset of TS					
<= 4	261	(25.1)	922	(24.8)	.030
5 to 7	517	(49.7)	1,709	(45.9)	
8 or Older	263	(25.3)	1,089	(29.3)	
Age First Seen					
<= 17	1,071	(87.4)	3,118	(74.6)	<.001
>17	154	(12.6)	1,063	(25.4)	
Age Diagnosed					
<= 8	416	(35.8)	1,275	(32.5)	<.001
9 to 12	488	(42.0)	1,366	(34.8)	
13 or Older	257	(22.1)	1,284	(32.7)	
Perinatal Problems					
Yes	280	(26.9)	607	(17.1)	<.001
No	761	(73.1)	2,945	(82.9)	
Has a Child with Tics					
Yes	12	(1.0)	104	(2.6)	.002
No	1,189	(99.0)	3,913	(97.4)	
At Least One Family Member with Tics					
Yes	613	(51.0)	2,201	(54.8)	.024
No	588	(49.0)	1,816	(45.2)	
Two or More Family Members with Tics					
Yes	145	(12.1)	609	(15.2)	.009
No	1,056	(87.9)	3,408	(84.8)	
Has Severe Tics					
Yes	226	(18.3)	668	(15.9)	.046
No	1,009	(81.7)	3,544	(84.1)	

Missing data alters the row and column totals for some variables.

Perinatal problems were prevalent in 27 percent of those with TS + LD, and only 17 percent for those with TS - LD (p < .001). The proportion of subjects with TS + LD were somewhat less likely to have a child with tics (p = .02) or have at least one family member with tics or TS (12 percent) when compared to the proportion of subjects with TS - LD (15 percent, p = .009). The proportion of subjects with severe tics in the TS + LD group was only slightly higher (18%) compared to those with TS - LD (16%), p = .046.

Subjects with TS + LD were more likely to have one or more of the fourteen comorbid disorders and conditions in the dataset when compared to those subjects with TS - LD (Table 2). Increases in comorbid conditions ranged from 0.7 percent for (psychotic disorder, p = .037) to 28.9 percent for (ADHD, p < .001). The mean number of comorbidities for subjects with TS + LD was 3.9 (s.d. = 2.2) and for subjects with TS - LD was 2.8 (s.d. = 2.0).

**Table 2:** Between group comparisons in 5,450 people with Tourette Syndrome and comorbid learning disabilities (TS + LD) and Tourette Syndrome without learning disabilities (TS - LD).

	TS + LD		TS - LD		
	n	(%)	n	%	p
ADHD	990	(80.2)	2,161	(51.3)	<.001
Anger	570	(46.2)	1,424	(33.8)	<.001
Sleep	372	(30.2)	998	(23.7)	<.001
Mood	266	(21.5)	767	(18.2)	<.001
Social Skills	409	(33.1)	620	(14.7)	<.001
Anxiety	249	(20.2)	686	(16.3)	.002
Sexual Behavior	85	(8.1)	137	(4.0)	<.001
CD	250	(20.2)	504	(12.0)	<.001
Coprolalia	199	(16.1)	531	(12.6)	.002
Stutter	137	(11.1)	271	(6.4)	<.001
Neurologic	104	(8.4)	218	(5.2)	<.001
DevD	137	(11.1)	180	(4.3)	<.001
PDD	101	(8.18)	167	(4.0)	<.001
Psy	20	(1.6)	37	(0.9)	.037

Attention deficit-hyperactivity disorder (ADHD), conduct disorder (CD), obsessive compulsive disorder (OCD), obsessive compulsive behavior (OCB), developmental disorder (DevD), learning disability (LD), mental retardation (MR), pervasive developmental disorder (PDD), psychosis (Psy) and neurological abnormality (Neurologic).

ADHD was the most prevalent comorbid disorder for subjects with TS + LD. In this population, 58% (3151) of the TS children had ADHD and 31% (990) of these had LD. The potential impact of ADHD on LD either as a causal factor or as a confounder for the diagnosis of LD is demonstrated by the finding that only 11 % (245) of the 2299 TS children without ADHD had LD.

The variables from Table 1 and the total number of comorbidities were entered into a logistic regression model. The optimal prediction model for the TS + LD group was comprised of five variables (being seen for evaluation before 18, being male, having fewer family members with tics or TS, having perinatal problems, and having more comorbidities). The logistic model performance characteristics were accuracy 65.2% (model correctly classified 4,406 of 5,450 subjects), sensitivity 66.1% and specificity of 62.2%.

## Discussion

In a population of 5,450 subjects with TS, we found 1,235 subjects with comorbid LD (TS + LD). Using logistic regression, we produced a five variable model that accurately predicted group membership for 65.2% of the 5,450 subjects in this study. The model parameters were male gender, fewer affected family members, increased rates of pregnancy, labor and delivery complications, increased prevalence of comorbidities and younger age at diagnosis. The absolute differences in rates of comorbidities between the groups for individual variables were often small and as a result the differences may be of limited clinical relevance. However, the five variable model may well have relevance for risk assessment for clinicians caring for subjects with TS and possibly for healthcare policy makers as well. Confirmation of our estimates of the performance characteristics of this model as a screening tool would require further study in a clinical setting. However, the development of a screening tool would be beneficial since delayed identification of learning disabilities results in delayed initiation of intervention services and likely increases the educational difficulty experienced by a person with an unidentified learning disability [22, 32].

The etiology of learning disabilities and the appropriate conceptual view of these diverse disorders as comorbid disorders or as variably prevalent components of the broader TS phenotype has yet to be resolved [3, 9, 10, 14, 15, 33]. In this study, ADHD was the most prevalent comorbid disorder with TS occurring in 57.8% of subjects (n = 3151). In subjects with TS + LD, 80.2% also had a diagnosis of ADHD and in the TS - LD group, 51.3% had a diagnosis of ADHD. We found that 31% of subjects with ADHD also had a diagnosis of LD compared to only 11% in subjects with TS who did not have ADHD. Thus, the comorbidity rates in this study may not differ from those reported for ADHD and reading disorders alone [34, 35]. where the prevalence of comorbidity between reading disorders and ADHD is 25 to 40%. The increased rates of ADHD in the TS + LD group may have multiple explanations including the possibility that ADHD is a confounder and that most cases of LD in subjects with TS represent the additional impairments in learning from the ADHD. In which case LD is misdiagnosed or that ADHD is an important component in the causal chain for LD and that LD is very often under diagnosed in subjects with ADHD. Additional research is required to determine which, if either, of these possibilities is correct. Other data sets will likely be required to examine the role of ADHD on LD in subjects with TS and other combinations of LD, ADHD and TS.

### Limitations

We have defined the two groups used in this study by the presence or absence of a clinically defined learning disability. We are unable to determine the accuracy of the diagnosis for subjects in this study. In this study we did not have the data to restrict LD cases to a single set of criteria. For example, we did not count only cases meeting the discrepancy criteria, which is a widely used strategy for the diagnosis of LD in the United States. We are not aware of a single diagnostic schema with wider acceptance around the world than the DSM criteria. As a result the prevalence estimates in this study may be biased. This might alter the accuracy of prevalence estimates or the significance testing for some variables. However, given the effect sizes found here, the bias would have to be consistent and quite large to alter the primary results.

## Conclusion

We found the prevalence of LD to be increased in subjects with TS. Additional studies are required to improve our understanding of the etiologic factors resulting in the expression of the patterns of individual syndromal variability noted in this and in other studies. It would be of interest to examine hypotheses to determine if subjects with TS have different types of LD or have specific patterns of comorbidity with LD. However, developing test batteries for subjects from over 20 different languages, cultures and differing academic systems seems a formidable task.

Improved understanding of the factors associated with a later diagnosis of an LD may have important implications for prevention of secondary disabilities especially those which result from symptom expression prior to a diagnosis of either TS or of TS with comorbidity [22, 36]. This recognition may be years or in some cases decades delayed. Ongoing research is needed to identify appropriate medical, psychosocial or educational management strategies for the two broad groups discussed here in this paper.

## Competing interests

The author(s) declare that they have no competing interest.

## Authors' contributions

RF, LB, and JK designed the study, and RF developed and maintained the TIC Registry. MK completed the data analysis. RF, LB, MK and JK wrote the manuscript and contributed important intellectual content. All authors have read and approved the final manuscript.

## Pre-publication history

The pre-publication history for this paper can be accessed here:



## Supplementary Material

Additional File 1 *TIC *Data Entry Form. The TIC Consortium Data Entry Form is a standardized form used by each center to extract data elements from the record for submission to the consortium database. Click here for file
