# Supplementary material for: Tourette syndrome and learning disabilities
Source: BMC Pediatr. 2005 Sep 1;5:34. doi: 10.1186/1471-2431-5-34 (PMC1242237; doi:10.1186/1471-2431-5-34)
Supplement: Additional File 1 — TIC Data Entry Form. The TIC Consortium Data Entry Form is a standardized form used by each center to extract data elements from the record for submission to the consortium database. [file 1471-2431-5-34-S1.doc]

My file #: ________) ***TIC* Data Entry Form[[1]](#footnote-2)*** Site Code: **ND 01**

**ID:** ____-____ - _______ ____ ____ - _______ **Gender:** ❑ Male ❑ Female

*Initials (2 only) year - month - day* ***M****ale/****F****emale*

<-- Birthdate -->

**Preferred hand:** ❑ Right ❑ Left ❑ Ambidextrous

**Adopted?** ❑ (Check if yes) **One of multiple births (e.g., twins)?** ❑ (Check if yes)

**DATE SEEN:** 200__-___-___ *[200_-month-day]* **Age FIRST SEEN by me:** ____ years

**Age of TIC ONSET:** ___ years (or ❑ unknown) **Age at DIAGNOSIS:** ___ years (or ❑ unknown)

**Family history:** ❑ Tics/TS: **M P S C** / ❑ OCD: **M P S C** / ❑ OCB: **M P S C** / ❑ ADHD: **M P S C**

***CIRCLE those that apply:*** *(Codes:* ***M*** *= maternal side,* ***P*** *= paternal,* ***S*** *= sibling,* ***C*** *= child (of adult patient))*

**Peak tic severity** (ever): ❑ Mild ❑ Moderate ❑ Severe [Composite clinical judgment of

[Specific scale, if any: _________; score ____] severity, frequency, and interference]

**Abrupt onset or up-surge after infection** (ever): ❑ Yes ❑ No ❑ Uncertain/don’t know

**Medication *for tics*** (ever)? ❑ Yes ❑ No ❑ Uncertain If so, *which?* __________________

**Prenatal/perinatal problems (significant):** ❑ Yes ❑ No ❑ Uncertain/don’t know

**Other problems**: *Note:* below is a screening list, calling for clinical judgment as to the presence of a significant disorder or problem, even if not present currently (specify where appropriate):

| ***Diagnosis or Problem Type:*** | ***Check***  ***if YES*** | ***Drugs***  ***(ever)*** | ***Specify Details, or***  ***"Other" Diagnoses:*** |
| --- | --- | --- | --- |
| Attention-Deficit/Hyperactivity Disorder | ❑ | ❑ |  |
| Obsessive-Compulsive *Disorder* | ❑ | ❑ | NOTE: not *both* Disorder & Behaviour |
| Obsessive-Compulsive *Behaviour* | ❑ | ❑ | NOTE: not *both* Disorder & Behaviour |
| Learning or Language Disorder | ❑ |  |  |
| Mood Disorder *(specify which)* | ❑ | ❑ |  |
| Anxiety Disorder *(specify which)* | ❑ | ❑ |  |
| Conduct Disorder | ❑ | ❑ |  |
| Oppositional Defiant Disorder | ❑ | ❑ |  |
| Pervasive Developmental Disorder:  **circle:** *autism, Asperger, Rett, PDD-NOS* | ❑ | ❑ |  |
| Psychotic Disorder *(specify which)* | ❑ | ❑ |  |
| Mental Retardation *(specify severity)* | ❑ |  |  |
| Eating Disorder *(specify which)* | ❑ |  |  |
| Developmental Disorder (other) | ❑ |  |  |
| Neurological Disorder *(specify)* | ❑ |  |  |
| Other Psychiatric Disorder *(specify)* | ❑ | ❑ |  |
| Other Medical Disorder *(specify)* | ❑ |  |  |
| Sleeping problems *any time after age 2* | ❑ | ❑ |  |
| Sleeping problems *(now, too)* | ❑ | ❑ |  |
| Anger control problems *any time after age 3* | ❑ | ❑ |  |
| Anger control problems *(now, too)* | ❑ | ❑ |  |
| Stuttering *any time after age 3* | ❑ | ❑ |  |
| Trichotillomania (hair-pulling) | ❑ | ❑ |  |
| Coprolalia/copropraxia *(specify)* | ❑ | ❑ |  |
| Self-injurious behavior *(specify)* | ❑ | ❑ |  |
| *Age (years) at onset of SIB* *[ASK!]* | ___ |  | *Be sure to ask date of onset of SIB* |
| Significant social skills problems | ❑ |  |  |
| Sexually inappropriate behavior *(specify)* | ❑ | ❑ |  |

**This date:** 2004-____-____ V*ersion of 2004-07-18;* ***discard any previous versions***

*month - day* Formerly the *"CATS"* Database *Copyright © Roger Freeman, 2004 - All rights reserved*

***NOTICE:*** *Avoid the most common error!* ***Age at onset****, and* ***Age at diagnosis*** *should be at least 1 year apart.*

1. ^*^***T***ourette syndrome ***I***nternational database ***C***onsortium. Enter only those cases meeting 1993 *Archives of Neurology* criteria. [↑](#footnote-ref-2)
